# Supplementary material for: Interlayer and Intralayer Excitons in AlN/WS2 Heterostructure
Source: Materials (Basel). 2022 Nov 23;15(23):8318. doi: 10.3390/ma15238318 (PMC9735989; doi:10.3390/ma15238318)
Supplement: Supplementary file 1 [file materials-15-08318-s001.zip › materials-1990183-supplementary.pdf]

Supplementary Material

Interlayer and intralayer excitons in AlN/WS_2 heterostructure

(Dated: November 14, 2022)

I. PROJECTED DENSITY OF STATES

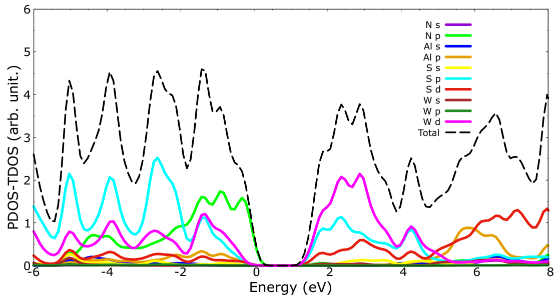

Fig. S1. AlN/WS_2 total and the projected density of states calculated within DFT approach using a $(48 \times 48 \times 1)$ **k**-point mesh and 400 bands. A broadening of 0.1 eV is applied.

II. EFFECT OF STRAIN

We considered two configuration for the heterostructure AlN/NWS_2: in the first configuration called *relaxed* we optimize the lattice parameters by minimizing the energy as explained in the main manuscript; in the second called *strained* we kept the lattice parameter equal to the one of isolated WS_2, a=3.11 Å. In Fig. S2 we report the GW band structure for the strained and relaxed structures. In Fig. S3 we report also the DFT band structure for the strained and relaxed AlN/WS_2. As one can see from this figure the bands inversion is complitely due to the GW correction and not the the strain.

We found that the direct gap at K changes from 2.80 eV to 2.71 eV, while the indirect remains more or less the same, see table S1.

conf.	alat Å	Direct Gap	Indirect Gap	Exciton binding
WS_2 eq	3.16	2.76	2.60	0.64
AlN/WS_2 strained	3.16	2.71	2.56	0.59
AlN/WS_2 relaxed	3.142	2.80	2.56	0.59

Table S1. Direct and indirect band gaps in eV (G_0W_0 level), first exciton energy for the different configurations.

Then we studied the optical response. In table S1 we reported the excitonic binding energy in the two structure strained and relaxed. We found that excitonic binding en-

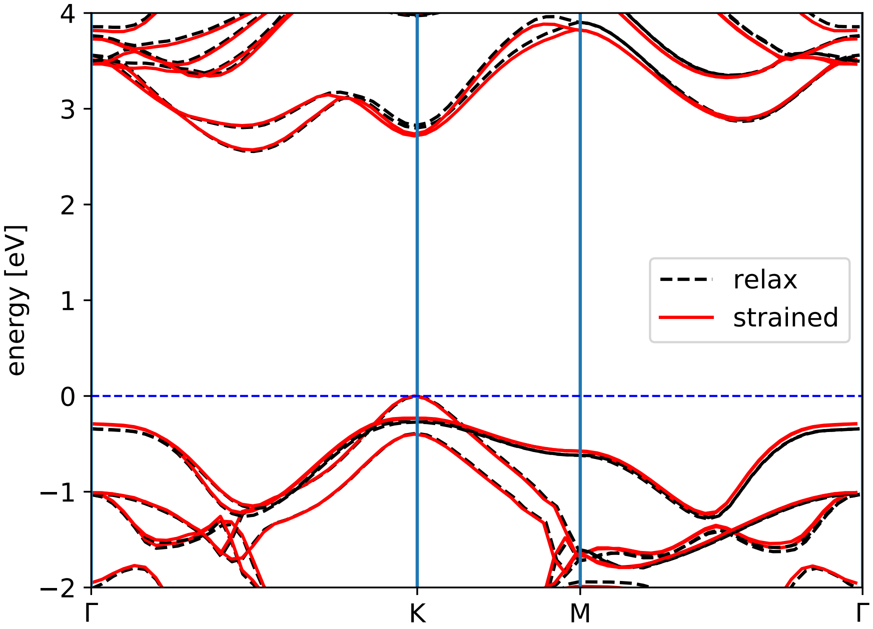

Fig. S2. GW band structure of $AlNWS_2$ relaxed and strained with the lattice parameter of the isolated WS_2.

ergy is unfected with the calculation precision from the strain, as it was found in other publications.[1]

[1] P. Lechifflart, F. Paleari, and C. Attaccalite, SciPost Physics **12**, 145 (2022).

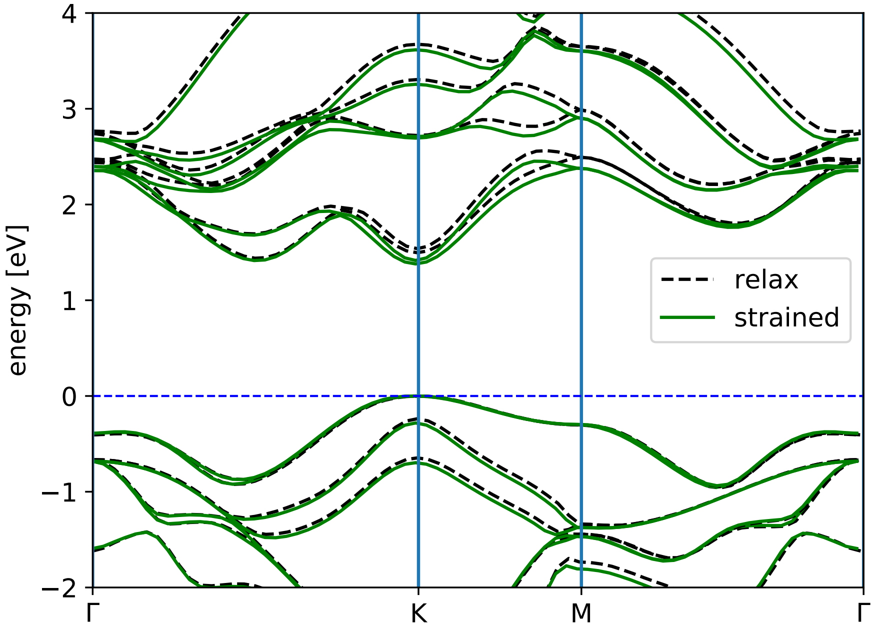

Fig. S3. DFT band structure of $AlNWS_2$ relaxed and strained with the lattice parameter of the isolated WS_2.

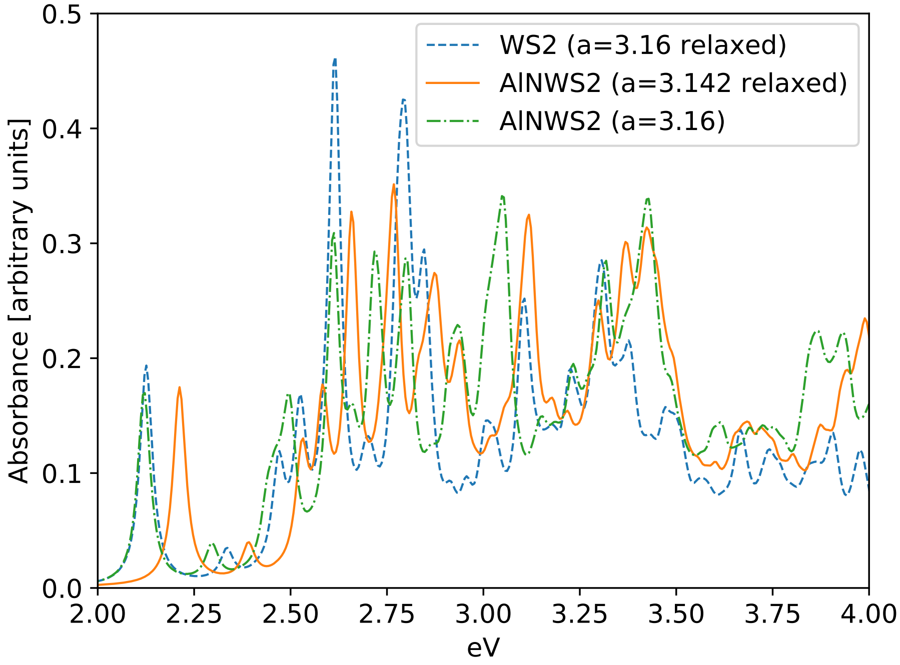

Fig. S4. Absorbance of AlN/WS2 heterostructure, calculated for the relaxed heterostructure and the one with the lattice parameter of the WS_2. For comparison we report also the absorbance of the isolated WS_2.
